# Oxidative stress in children late after Kawasaki disease: relationship with carotid atherosclerosis and stiffness

**DOI:** 10.1186/1471-2431-8-20

**Published:** 2008-05-08

**Authors:** Yiu-fai Cheung, Karmin O, Connie WH Woo, Stephanie Armstrong, Yaw L Siow, Pak-cheong Chow, Eddie WY Cheung

**Affiliations:** 1 Division of Paediatric Cardiology, Department of Paediatrics and Adolescent Medicine, Grantham Hospital, The University of Hong Kong, Hong Kong, China; 2 Canadian Centre for Agri-Food Research in Health and Medicine, Department of Animal Science, University of Manitoba, Winnipeg, Canada; 3 Department of Physiology, University of Manitoba, Winnipeg, Canada

## Abstract

**Background:**

Persistent arterial dysfunction in patients with a history of Kawasaki disease (KD) and an integral role of oxidative stress in the development of cardiovascular disease are increasingly recognized. We sought to test the hypothesis that oxidative stress is increased in KD patients and related to carotid atherosclerotic changes and stiffness.

**Methods:**

We compared the serum levels of oxidative stress biomarkers, carotid intima-media thickness (IMT), and carotid stiffness index among KD patients with coronary aneurysms (n = 32), those without coronary complications (n = 19), and controls (n = 32).

**Results:**

Compared with controls, patients with coronary aneurysms had significantly higher serum levels of malonaldehyde (2.62 ± 0.12 μM *vs *2.22 ± 0.07 μM, p = 0.014) and hydroperoxides (26.50 ± 1.13 μM *vs *22.50 ± 0.62 μM, p = 0.008). A linear trend of the magnitude of oxidative stress in relation to inflammatory damage was observed for malonaldehyde (p = 0.018) and hydroperoxides (p = 0.014) levels. Serum malonaldehyde and hydroperoxide levels correlated positively with carotid IMT (p < 0.001 and p = 0.034, respectively) and stiffness index (p = 0.001 and p = 0.021, respectively). Multiple linear regression analysis identified serum malonaldehyde level as a significant determinant of carotid IMT (β = 0.31, p = 0.006) and stiffness (β = 0.27, p = 0.008).

**Conclusion:**

Our findings suggest oxidative stress is increased in KD patients with coronary aneurysms and is associated with carotid intima-media thickening and stiffening.

## Background

Since its first description in 1967 [1], Kawasaki disease (KD) has become the most common acquired heart disease in children in developed countries [2]. Patients diagnosed with KD in the sixties and early seventies have reached middle age. Previous studies have demonstrated persistent coronary arterial dysfunction in patients with persistent and regressed coronary aneurysm [3-5]. Importantly, endothelial dysfunction and stiffening of both the coronary [3-5] and systemic [6-9] arteries that characterize the arterial dysfunction are themselves risk factors for premature atherosclerosis and cardiovascular disease. Indeed, we [10] and others [7] have found a significant increase in carotid intima-media thickness (IMT), a marker of atherosclerosis [11], in KD patients with or without coronary complications late after the acute illness. While growing data suggest premature atherosclerosis in patients with a history of KD, the underlying mechanisms remain unclear.

Accumulating evidence suggests that oxidative stress plays an integral role in the development of atherosclerosis and cardiovascular disease. Reactive oxygen species are increasingly recognized to play an important role in compromising endothelial function, including modulation of vasomotor tone [12,13]. Oxidized low-density lipoprotein is pro-inflammatory [14,15], causes inhibition of endothelial nitric oxide synthase [16], promotes retention of macrophages in the arterial wall [17], and stimulates vascular smooth muscle cells proliferation [15]. Furthermore, F2-isoprostanes, a biomarker marker of lipid peroxidation [18], have been found to localize in foam cells in atherosclerotic lesions of humans [19] and a mouse model of atherosclerosis [20].

Given the persistent arterial dysfunction and risk of premature atherosclerosis in KD patients as well as the increasingly recognized integral role of oxidative stress in the development of cardiovascular disease, we hypothesized that oxidative stress was increased in KD patients and related to carotid atherosclerotic changes and stiffness. To test the hypothesis, we compared the levels of two markers of oxidative stress, malonaldehyde and hydroperoxides, in patients with a history of KD with those of healthy controls and determined the correlations between levels of these oxidative stress markers and carotid IMT and stiffness index.

## Methods

### Subjects

Patients with a history of KD were recruited from the paediatric cardiac clinic. Those diagnosed with KD within 12 months of study were excluded to minimize potential confounding influence relating to subacute inflammation. From the case records, the age at diagnosis, the interval from disease onset to the time of study, and the coronary artery status were noted. Coronary aneurysms were documented by serial two-dimensional echocardiography in all of the patients. None of the patients required cardiac catheterization. Diagnosis of KD was based on the classic clinical criteria [21], while diagnosis of coronary aneurysms was based on the Japanese Ministry of Health criteria [22]. Healthy age-matched subjects were recruited as controls were recruited as control subjects. These were healthy children previously discharged from our clinic with a diagnosis of a functional heart murmur and their healthy siblings. The body weight and height were measured, and body mass index was calculated accordingly. The blood pressure in the right arm was measured twice using an automated oscillometric device (Dinamap, Critikon, Inc), and the average of the 2 readings was taken. The institutional Ethics Committee approved the study and parents of all subjects gave written, informed consent.

### Measurement of carotid IMT and stiffness

A 7–15 MHz linear-array transducer, interfaced to a Hewlett-Packard Sonos 5500 ultrasound machine, was used to image the right carotid artery at about 1 cm proximal to the carotid bifurcation. The IMT of the right common carotid artery far wall was measured offline using the QLAB analysis software (Philips, Bothell, USA). The QLAB IMT plug-in detects automatically the intima-media pixel pairs along the far wall of the carotid artery and provides a spatially averaged measurement of IMT over a preset of 10 mm region of interest. The average of three measurements was used for subsequent analysis.

The carotid artery stiffness was assessed by calculating the stiffness index as reported previously [23]. The end-diastolic (Dd) and systolic (Ds) diameters were measured. The carotid arterial stiffness index was then calculated as: ln (SBP/DBP)/[(Ds-Dd)/Dd], where SBP and DBP are systolic and diastolic blood pressure, respectively. The average of the right carotid arterial stiffness index derived from the values of three consecutive cardiac cycles was used for subsequent analyses.

### Blood investigations

Venous blood was withdrawn for measurement of fasting lipid profiles, malonaldehyde and hydroperoxide levels in patients and controls. Serum total cholesterol level was determined enzymatically using a Hitachi 912 analyzer (Roche Diagnostics, GmbH, Mannheim, Germany). High-density lipoprotein (HDL) cholesterol was measured using a homogenous method with polyethylene glycol modified enzymes and sulphated α-cyclodextrin. Low-density lipoprotein (LDL) cholesterol was calculated by the Friedewald equation.

Under oxidative stress, lipid peroxidation serves as an important contributor to cell injury. The degree of lipid peroxidation in plasma was determined by measuring thiobarbituric acid reactive substances (TBARS) [24,25]. In brief, an aliquot of serum (100 μL) was added to 10% phosphotungstic acid (500 μL) and incubated at room temperature for 10 minutes. The mixture was centrifuged at 2000 *g *for 10 minutes. After removal of supernatant, thiobarbituric acid (0.67%) was added to the pellet and incubated at 95°C for 1 hour. The amount of malonaldehyde formed in the reaction mixture was measured by spectrophotometer at absorbance of 532 nm. Malonaldehyde was used as the standard. The amount of malonaldehyde detected correlates to the level of lipid peroxides in the serum.

Serum hydroperoxide levels were measured by modified FOX assay [26,27]. In brief, an aliquot of plasma (100 μL) was added to FOX2 reagent and incubated at room temperature for 30 minutes. The reaction mixture was then centrifuged at 12,000 g for 5 minutes. Absorbance (at 560 nm) of the supernatant was measured. Hydrogen peroxide was used as standard.

### Data analysis

Data are presented as mean ± SEM. Differences in demographic data between patients and control subjects were compared using unpaired Student's *t *test. The malonaldehyde and hydroperoxides levels among patients with coronary aneurysms, patients without coronary complications, and control subjects were compared using simple analysis of variance, with posthoc comparisons by Tukey test. For the entire cohort, Pearson correlation analysis was used to assess for possible relationships between levels of oxidative stress markers and carotid IMT and stiffness index. Stepwise multiple linear regression was used to identify significant determinants of carotid IMT and stiffness. A p value of less than 0.05 was considered statistically significant. All statistical analyses were performed using SPSS version 10.0 (SPSS, Inc., Chicago, Illinois).

## Results

A total of 83 Chinese patients living in Hong Kong were studied, comprising 51 patients with KD and 32 controls subjects. Those patients were studied at 10.5 ± 0.68 years after initial diagnosis of KD. Of the 51 patients, 32 had coronary aneurysms, which were persistent in 26 and had regressed in 6. All of the patients were asymptomatic, none required coronary arterial interventions, and 26 patients whom had persistent coronary aneurysms were maintained on long-term aspirin therapy. None of the patients were smokers and none had family history of premature cardiovascular disease. Table 1 shows the demographic and clinical data of the patients and controls. There were no significant differences in age, sex distribution, body mass index, blood pressure and cholesterol levels between patients and controls.

**Table 1:** Demographic data, blood pressure and cholesterol levels of patients and controls

	Patients (n = 51)	Controls (n = 32)	p
Age (yrs)	13.4 ± 0.6	14.6 ± 0.6	0.18
Gender (M/F)	40/11	26/6	1.00
BMI (kg/m^2^)	19.5 ± 0.6	19.8 ± 0.6	0.71
Systolic blood pressure (mmHg)	112 ± 1.7	113 ± 2.2	0.61
Diastolic blood pressure (mmHg)	65 ± 1.1	66 ± 1.1	0.79
Total cholesterol (mmol/L)	4.09 ± 0.9	4.09 ± 0.1	0.99
HDL cholesterol (mmol/L)	1.11 ± 0.03	1.16 ± 0.03	0.32
LDL cholesterol (mmol/L)	2.64 ± 0.08	2.65 ± 0.1	0.95

BMI = body mass index; HDL = high-density lipoprotein; LDL = low-density lipoprotein

**Figure 1:**
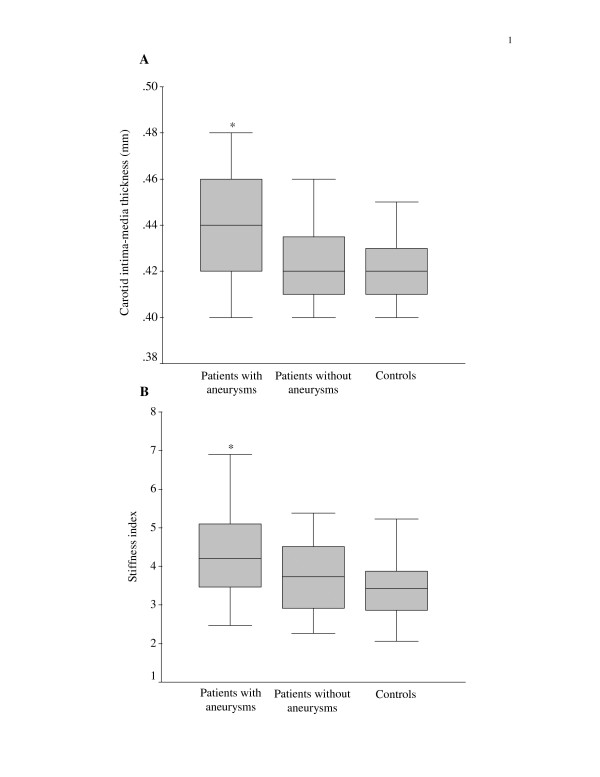
Box-plots showing (A) carotid intima-media thickness, and (B) stiffness index in patients and controls (*p < 0.05 when compared with control group).

Compared with controls, patients with coronary aneurysms had significantly greater carotid IMT than those without aneurysm (p = 0.024) and controls (p = 0.006) (Figure 1a). Likewise, the stiffness index was greater in patients with than those without coronary aneurysm (p = 0.088) and controls (p < 0.001) (Figure 1b).

**Figure 2:**
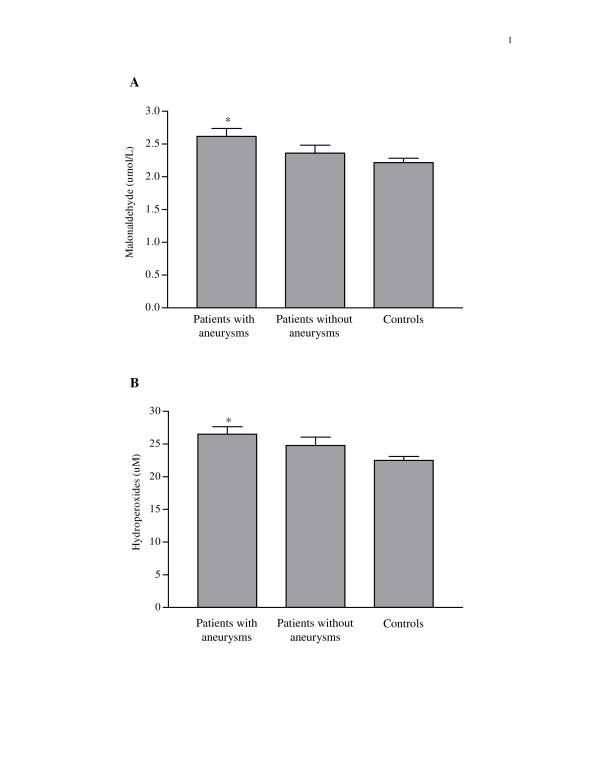
The serum levels of (A) malonaldehyde and (B) hydroperoxides in patients with coronary aneurysms, patients without coronary aneurysms, and controls (*p < 0.05 when compared with control group).

The serum levels of malonaldehyde and hydroperoxides, biomarkers of oxidative stress, in patients with or without coronary aneurysm and controls are shown in Figure 2. Compared with controls, patients with coronary aneurysms had significantly higher levels of malonaldehyde (2.62 ± 0.12 μM *vs *2.22 ± 0.07 μM, p = 0.014) and hydroperoxides (26.50 ± 1.13 μM *vs *22.50 ± 0.62 μM, p = 0.008). By contrast, the levels of malonaldehyde (2.58 ± 0.15 μM *vs *2.77 ± 0.17 μM, p = 0.22) and hydroperoxides (27.2 ± 1.2 μM *v*s 23.3 ± 3.2 μM) were similar between patients with persistent aneurysm and those with regressed ones.

The possibility of a linear trend of the magnitude of oxidative stress was further determined using linear regression analysis of in the three cohorts: patients with coronary aneurysms, patients without coronary aneurysms, and controls. A significant trend was observed for malonaldehyde (*P *= 0.018) and hydroperoxides (p = 0.014) levels.

**Figure 3:**
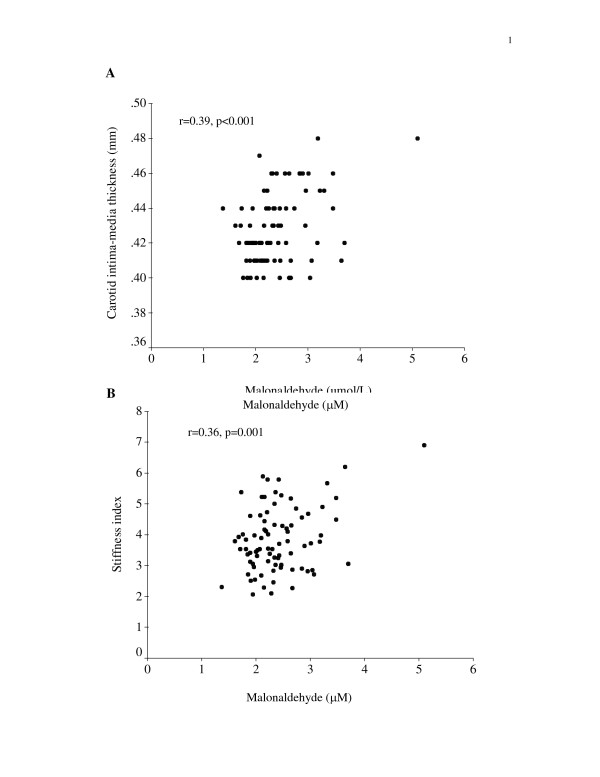
Scatter plots showing positive correlations between malonaldehyde level and (A) carotid intima-media thickness, and (B) stiffness index.

**Figure 4:**
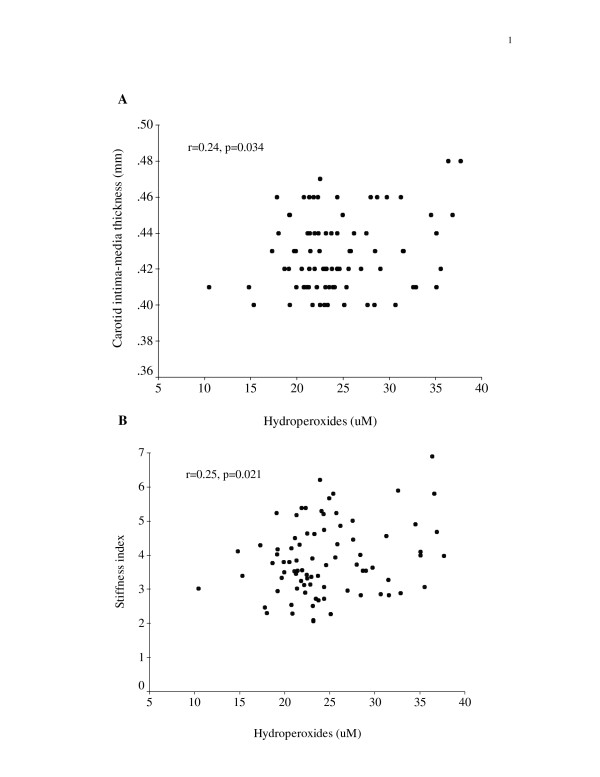
Scatter plots showing positive correlations between hydroperoxide level and (A) carotid intima-media thickness, and (B) stiffness index.

Correlations between levels of oxidative stress markers and carotid IMT and stiffness index are shown in Figures 3 and 4. The malonaldehyde level correlated positively with both the carotid IMT (p < 0.001) and stiffness index (p = 0.001). Similarly, the hydroperoxide level correlated positively with the carotid IMT (p = 0.034) and stiffness index (p = 0.021).

Multiple linear regression analysis of the entire cohort was used to identify significant determinants of carotid IMT and stiffness index. The independent variables included age, sex, body mass index, subject grouping (patients with coronary aneurysms as the referent group with 2 dummies for patients without coronary complications and controls), systolic and diastolic blood pressures, total cholesterol, LDL and HDL cholesterols, and malonaldehyde and hydroperoxides level. Significant determinants of carotid IMT were subject grouping (β = -0.24, p = 0.025) and malonaldehyde level (β = 0.31, p = 0.006) (model R^2 ^= 0.20), while significant determinants of carotid stiffness were subject grouping (β = -0.35, p = 0.001), body mass index (β = 0.26, p = 0.01), and malonaldehyde level (β = 0.27, p = 0.008) (model R^2 ^= 0.29).

## Discussion

The novel findings of the present study are (1) a significant increase in the plasma levels of oxidative stress markers late after the acute illness in patients with a history of KD and coronary aneurysms and (2) positive correlations between markers of oxidative stress and carotid IMT and stiffness. As one of the most sensitive targets for attacks of oxidative stress are unsaturated fatty acids of cellular lipid membranes, we measured the levels of hydroperoxides and malonaldehyde, the latter being one of the widely used indices of oxidative stress.

Previous studies have provided clues for possible existence of increased oxidative stress in patients after KD. Deng and colleagues have demonstrated the restoration of systemic arterial endothelial dysfunction in patients with and without coronary aneurysms at 1 to 10 years after initial diagnosis of KD by acute intravenous administration of vitamin C [28]. They hypothesized that the antioxidant action of vitamin C might be responsible for the beneficial effect [29]. Furthermore, endothelin-1, which could induce superoxide production [30], has been shown to be increased in KD in the plasma in the acute and recovery stage [31]. To the best of our knowledge, the present study is the first to provide direct evidence of increased oxidative stress in KD patients with coronary aneurysms, which shed light on the possible underlying mechanisms of the previously reported, but hitherto unexplained, arterial dysfunction and intima-media thickening in these patients.

Arterial dysfunction is increasingly recognized as a long-term sequel of KD, even in patients without coronary aneurysms [3-8]. Impaired coronary vasodilatory response to intracoronary injection of acetylcholine [4,5] and nitroglycerine [3,5] has been demonstrated at sites of persistent and regressed aneurysms. Concerns have also been raised with regard to the possibility of impaired acetylcholine-induced vasodilation at sites with apparently normal coronary arteries [32]. On the other hand, impaired brachial flow-mediated dilation [6] and increased conduit arterial stiffness [8] have been shown in KD patients with and without coronary complications. Importantly, endothelial dysfunction and arterial stiffening that characterize the arterial abnormalities in KD patients are risk factors for cardiovascular disease and atherosclerosis. Over the past two decades, accumulating evidence suggests that endothelial dysfunction may be caused by accelerated inactivation of nitric oxide by reactive oxygen species, which reduces bioavailability of nitric oxide [33]. In adult patients with coronary artery disease [34], non-insulin-dependent diabetes mellitus [35], and hypertension [36], administration of vitamin C has similarly been shown to improve endothelial dysfunction. While vascular damage with consequent structural alteration might in part account for arterial stiffening in patients with a history of KD [8], the alteration of vasomotor tone secondary to endothelial dysfunction might also be a culprit [6]. The association between oxidative stress and arterial elasticity has recently also been demonstrated by Kals and coworkers in adult patients with peripheral arterial disease [37]. In their patients, inverse associations exist between large and small artery elasticity and urinary 8-iso-prostaglandins F_2α _generated as a result of free radical-mediated peroxidation of arachidonic acid [38]. Proposed mechanisms include modulation of matrix metalloproteinases associated with destruction of arterial elastic laminae [39], enhancement of smooth muscle tone [40], and promotion of smooth muscle cell proliferation [41] by reactive oxygen species. In adults with renovascular hypertension, an inverse relationship between urinary excretion of 8-hydroxy-2'-deoxygnanosine and acetylcholine-induced forearm blood flow has been reported [42]. A recent study has further provided evidence that local oxidative stress as assessed by net production of isoprostane across the left anterior descending artery territory has a role in the reduction of nitric oxide bioavailability in humans with coronary endothelial dysfunction [43]. Hence, it is tempting to speculate that oxidative stress in KD patients with coronary aneurysms as shown in the present study might in part account for the observed long-term systemic arterial dysfunction, albeit the latter is being documented also in patients without coronary complications [6,8,28].

The next logical question to address is the source of oxidative stress in KD patients. We [44] and others [45] have shown that high-sensitivity C-reactive protein is elevated in KD patients with coronary aneurysms late after the acute illness and implicate the persistence of chronic low-grade inflammation in these patients. C-reactive protein has been reported to induce superoxide production in human aortic endothelial cells as well as in smooth muscle cells [46], possibly through activation of vascular nicotinamide adenine dinucleotide/nicotinamide adenine dinucleotide phosphate oxidase system [47]. The association of inflammation and increased oxidative stress amplifies the cascade that results in vascular damage [48]. Depletion of L-arginine in inflammatory sites has further been shown to trigger inducible nitric oxide synthase (iNOS) in macrophages to generate functionally important amounts of superoxide anion [49]. Indeed, we have previously demonstrated that the serum of patients with a history of KD induces expression iNOS in THP-1 macrophages in vitro [50]. Although the role of iNOS in atherosclerosis remains to be defined, genetic deficiency in iNOS has been reported to result reduce atherosclerosis in apolipoprotein E-deficient mice [51] and that chronic treatment of iNOS inhibitors has been shown to limit progression of preexisting atherosclerosis in hypercholesterolaemic rabbits [52]. Undoubtedly, these speculations of potential sources of oxidative stress in KD patients require further studies for clarification.

Our findings of increased oxidative stress in KD patients and its relation with arterial stiffening and intima-media thickening have important therapeutic implications. Notwithstanding the reported acute improvement of endothelial function with intravenous vitamin C administration in KD patients [28], the usefulness of antioxidant vitamins to prevent cardiovascular disease in adults with and without hypercholesterolaemia, end-stage renal disease, atherosclerosis has been controversial [53,54]. Further studies are necessary to elucidate the optimum dose and type of antioxidants and the impact of long-term antioxidant supplementation on the arterial IMT and stiffness in KD patients.

A limitation to this study is the potential confounding influence of aspirin prescribed to KD patients with coronary aneurysms on the levels of oxidative stress markers. Nonetheless, in a rat model, aspirin has been shown to attenuate the elevation in aortic superoxide anion levels induced by angiotensin II [55]. Thus, in patients with persistent coronary aneurysms, if aspirin were to have any influence on the levels of oxidative markers, the chance of detecting a significant difference among groups would be minimized rather than exaggerated.

## Conclusion

Increased oxidative stress occurs in patients with a history of KD late after the acute illness and is associated with arterial stiffening and intima-medial thickening. Further research to determine the potential benefits of antioxidants in these patients at risk of premature atherosclerosis and cardiovascular disease is warranted.

## Competing interests

The authors declare that they have no competing interests.

## Authors' contributions

YFC and KO participated in the design of the study, analysis of data, and drafting of the manuscript. CWHW, SA, and YLS performed the laboratory assay of oxidative stress markers and participated in statistical analysis of laboratory results, while PCC and EWYC coordinated the study of patients and performed the echocardiographic studies. All authors have read and approved the final manuscript.

## Pre-publication history

The pre-publication history for this paper can be accessed here:


